# Contemporary Diagnosis and Management of Hypertrophic Cardiomyopathy: The Role of Echocardiography and Multimodality Imaging

**DOI:** 10.3390/jcdd9060169

**Published:** 2022-05-25

**Authors:** Takeshi Kitai, Andrew Xanthopoulos, Shoko Nakagawa, Natsuko Ishii, Masashi Amano, Filippos Triposkiadis, Chisato Izumi

**Affiliations:** 1 National Cerebral and Cardiovascular Center, Department of Cardiovascular Medicine, Suita 564-8565, Japan; shoko-nakagawa1219@ncvc.go.jp (S.N.); ishii.natsuko@ncvc.go.jp (N.I.); m.amano@ncvc.go.jp (M.A.); izumi-ch@ncvc.go.jp (C.I.); 2 Department of Cardiology, University Hospital of Larissa, 41110 Larissa, Greece; andrewvxanth@gmail.com (A.X.); ftriposkiadis@gmail.com (F.T.)

**Keywords:** hypertrophic cardiomyopathy, echocardiography, multimodality imaging

## Abstract

Hypertrophic cardiomyopathy (HCM) is an underdiagnosed genetic heart disease with an estimated prevalence of 0.2–0.5%. Although the prognosis of HCM is relatively good, with an annual general mortality of ~0.7%, some patients have an increased risk of sudden death, or of developing severe heart failure requiring heart transplantation or left ventricular (LV) assist device therapy. Therefore, earlier diagnosis and proper identification of high-risk patients may reduce disease-related morbidity/mortality by promoting timely treatment. Echocardiography is the primary imaging modality for patients with suspected HCM; it plays central roles in differential diagnosis from other causes of LV hypertrophy and in evaluating morphology, hemodynamic disturbances, LV function, and associated valvular disease. Echocardiography is also an essential tool for the continuous clinical management of patients with confirmed HCM. Other imaging modalities, such as cardiac computed tomography (CT) and cardiac magnetic resonance imaging (MRI), can supplement echocardiography in identifying high-risk as well as milder HCM phenotypes. The role of such multimodality imaging has been steadily expanding along with recent advancements in surgical techniques and minimally invasive procedures, and the emergence of novel pharmacotherapies directly targeting pathogenic molecules such as myosin inhibitors. Here we review essential knowledge surrounding HCM with a specific focus on structural and functional abnormalities assessed by imaging modalities, leading to treatment strategies.

## 1. Introduction

Hypertrophic cardiomyopathy (HCM) is a common inherited heart disease with an estimated prevalence ranging from 0.2 to 0.5% based on echocardiography screening [1,2,3,4,5], and it is thought that many patients are underdiagnosed [6,7]. HCM is caused by mutations of sarcomeric genes with heterogeneous clinical phenotypes, and its pathological hallmarks include non-physiological left ventricular (LV) hypertrophy not related to pressure or volume overload [1,8]. Although disease severity varies from asymptomatic or fairly symptomatic to advanced heart failure and fatal ventricular arrhythmia leading to sudden cardiac death (SCD), the general prognosis of HCM is relatively good, with a general mortality rate of about 0.7% [9,10,11]. Nevertheless, some patients have increased risks of SCD or of developing severe heart failure requiring heart transplantation or LV assist device therapy. Therefore, the identification of such high-risk patients is a critical issue [1,8], and earlier and prompt diagnosis leading to prognostic stratification may reduce disease-related complications by promoting optimal management. Recent advancements in the identification of pathogenic mutations in HCM have provided a new patient spectrum of genotype-positive relatives who do not have signs and symptoms of HCM. Several studies have reported that these patients have subtle abnormalities in the mitral valve apparatus or LV function. 

Echocardiography is the primary modality for suspected HCM and plays a central role in excluding other causes of LV hypertrophy and in evaluating morphology, hemodynamic disturbances, and LV function [12,13]. Repeat echocardiography is also an important tool for the continuous clinical management of patients with HCM and for identifying markers associated with worse prognosis. Because preventing SCD is one of the important targets in the management of HCM, a continuous effort has been made to identify echocardiographic parameters related to SCD. The current SCD risk calculators endorsed by the European Society of Cardiology (ESC) and the American Heart Association (AHA) include echocardiographic measurements [1,8]. Recently, other imaging modalities, including cardiac computed tomography (CT) and cardiac magnetic resonance imaging (MRI), have been employed to supplement and support echocardiography with the identification of the high-risk HCM phenotype as well as with the identification of milder spectrum or early stages of HCM. In addition, the role of multimodality imaging has been steadily expanding, with recent advancements in medical therapy directly targeting pathogenic molecules. In this article, we review essential knowledge of HCM, with a specific focus on structural and functional abnormalities assessed by imaging modalities, leading to treatment strategies.

## 2. Diagnosis and Variation of Hypertrophy

The diagnostic criteria for HCM are currently as follows: prominent LV hypertrophy without other causes of hypertrophy, a maximum LV wall thickness of ≥15 mm, or a maximum LV wall thickness of ≥13 mm for patients with a first-degree relative with confirmed HCM [1,8]. The pattern and the distribution of LV hypertrophy are highly variable. The interventricular septum is commonly affected, but LV hypertrophy can also be isolated to the LV free wall, apex, and anterolateral wall. Asymmetric hypertrophy is considered one of the hallmarks of HCM, defined as a septal-to-posterior wall thickness ratio of ≥1.3 in normotensive patients or ≥1.5 in hypertensive patients. However, some patients with hypertension can have asymmetric hypertrophy, so this definition is not necessarily specific to HCM [13]. 

In addition to the hypertrophic LV wall, HCM is characterized by heterogeneous clinical expression, in which some patients may present with severe symptoms of dyspnea, chest pain, or syncope, while other patients may remain asymptomatic. Structural abnormalities, such as left ventricular outflow tract (LVOT) or mid-ventricular obstruction (MVO), left ventricular apical aneurysm, systolic anterior motion of the mitral valve, and other mitral valve apparatus abnormalities are often related to the symptoms and complications of HCM. 

### Assessment of LV Wall Hypertrophy 

Echocardiography is the primary imaging modality in the screening, diagnosis, prognostic stratification, and follow-up of HCM patients [1,8,14]. Comprehensive echocardiographic evaluations are recommended for the initial disease screening. As a first step for the diagnosis of HCM, careful assessment of LV wall thickness from the base to the apex is essential. LV wall thickness is recommended to be measured at end-diastole and preferably in parasternal short axis views, but it is important not to include RV trabeculations while measuring the septal wall thickness. The LV wall thickness measured at any site of the LV end-diastole is defined as maximal diastolic LV wall thickness. Although two-dimensional (2D) echocardiography is the first-line modality for diagnosing HCM, it may fail to diagnose some HCM phenotypes, such as localized hypertrophy of LV segments. Even localized mild hypertrophy is still at risk for disease complications like SCD. In patients with poor echocardiographic images, cardiovascular magnetic resonance (CMR) imaging is useful to detect the site and extent of LV hypertrophy. In addition to the LV wall, CMR can provide high-resolution moving images of systolic anterior motion (SAM) of the mitral valve, LVOT flow turbulence, mitral regurgitation, and abnormalities in papillary muscles.

**Figure 1:**
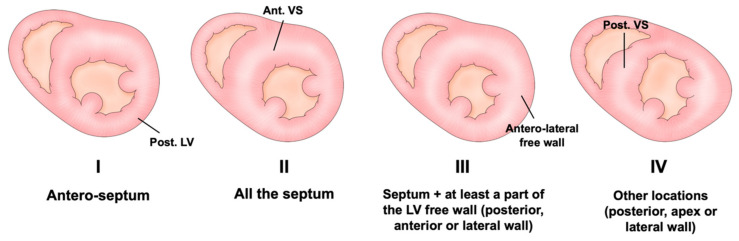
The classical four phenotypes of Maron’s classification based on the location and degree of hypertrophy. Post. = posterior; LV = left ventricle; Ant. = anterior; VS = ventricular septum.

**Figure 2:**
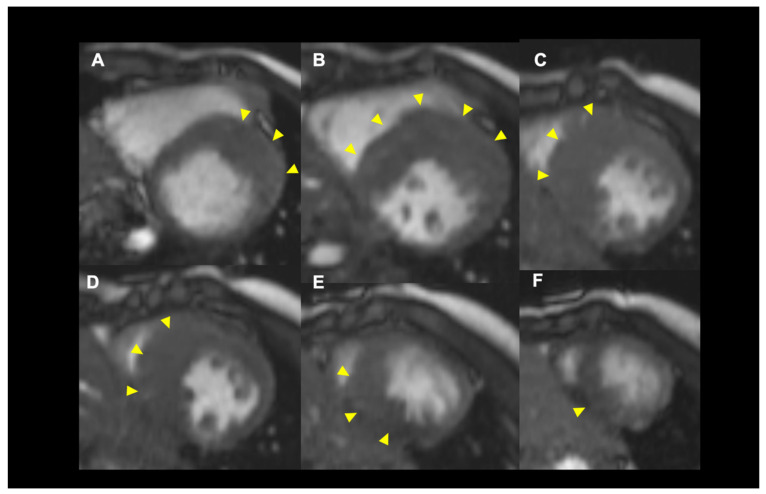
A representative case in which the hypertrophied region is changing along with the helical structure of myocardial fibers. The arrowheads indicate the location of hypertrophy. LV short axis views from the basal (**A**) to the apex (**F**).

The patterns of hypertrophy vary. Originally, Maron et al. proposed the following four-type classification based on the location of LV hypertrophy: type I, hypertrophy involving the basal anteroseptum; type II, hypertrophy involving the whole septum; type III, hypertrophy involving the whole septum and at least one region among the anterior, posterior, or lateral wall; and type IV, other localization involving the posterior, apex, or lateral wall (Figure 1) [15]. Although this classification is the most popular and clinically useful, the localization of the thickened wall changes depending on the cross section of the short axis view in echocardiography. This results from the helical arrangement of the muscle fibers in the LV (Figure 2). Syed et al. proposed the following five-phenotype classification: type A, a predominant mid-septal convexity toward the LV cavity, with the cavity often having a crescent shape (reverse curvature septum HCM); type B, a generally ovoid LV cavity, with the septum being concave to the LV cavity and a prominent basal septal bulge (sigmoid septum HCM); type C, an overall straight septum that is neither predominantly convex nor concave toward the LV cavity (neutral septum HCM); type D, predominant apical distribution of hypertrophy (apical HCM); and type E, predominant hypertrophy at the mid-ventricular level (mid-ventricular HCM) [16]. Helmy proposed another classification based on clinical presentation: pattern 1, septal hypertrophy alone; pattern 2, septum and adjacent segments’ hypertrophy, but not apical hypertrophy; pattern 3, apical in combination with other LV segments’ hypertrophy; and pattern 4, apical hypertrophy alone [17]. These differences are likely determined by the underlying genetic substrate, but the data are currently insufficient to confirm this hypothesis.

Although the diagnosis of HCM primarily depends on unphysiological LV hypertrophy, LV hypertrophy can also be caused by other conditions, such as hypertensive heart disease, athlete heart, aortic stenosis, cardiac amyloidosis, Fabry disease, and sigmoid septum leading to isolated basal hypertrophy. Although maximal LV wall thickness greater than 15 mm is favorable for the diagnosis of HCM and unusually observed in the other conditions, this is not specific for HCM. In addition to the clinical information such as symptoms, heart murmur, family history, and genetic testing, multimodality imaging is useful for the differential diagnosis. For example, a cardiac magnetic resonance late gadolinium enhancement imaging (CMR-LGE) pattern of patchy distribution in middle segment is a typical finding in HCM. On the other hand, low QRS voltage in ECG, apical sparing pattern in longitudinal strain echo imaging, and ^99m^Tc-pyrophosphate (PYP) scintigraphy are characteristics of the cardiac amyloidosis. Although differentiation from athlete’s heart is sometimes challenging, the absence of LGE and reduction in extracellular volume by T1 mapping in CMR are favorable for the diagnosis of athlete’s heart [18]. 

## 3. Various Types of Left Ventricular Structural Abnormality

### 3.1. Left Ventricular Outflow Obstruction (LVOTO)

**Figure 3:**
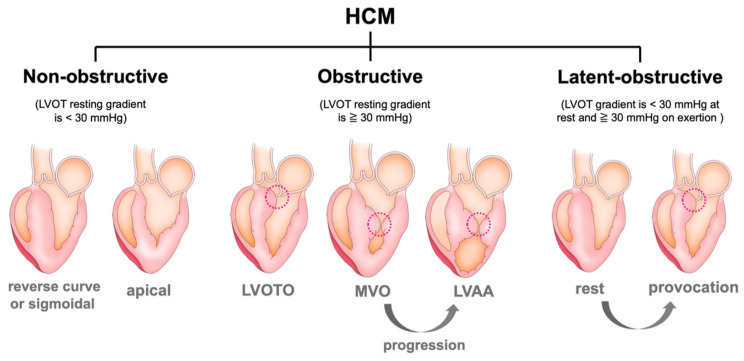
Schematic diagram of the different variants of HCM based on hemodynamic (obstructive vs. non-obstructive) classification. HCM = hypertrophic cardiomyopathy, LVOT = left ventricular outflow tract, LVOTO = left ventricular outflow tract obstruction, MVO = mid-ventricular obstruction, and LVAA = left ventricular apical aneurysm.

In addition to the aforementioned classifications, mainly based on the location of LV hypertrophy, functional and hemodynamical classifications also exist. An obstructive HCM is defined as a significant intraventricular pressure gradient of ≥30 mmHg. One-third of patients with HCM have significant obstructions at rest, one-third are latent obstructive after provocative maneuvers (Valsalva/standing/exercise), and one-third are truly non-obstructive (Figure 3) [8,19,20]. Intraventricular obstruction, such as that in the LVOT and mid-ventricle, is dynamic and may vary depending on loading conditions; additionally, it is associated with symptoms and heart failure progression even in patients with latent obstruction. Further, 38% of patients who have resting obstruction have symptoms, compared to 20% of patients with provocative obstruction and 10% of patients without obstruction [8]. LVOT obstruction (LVOTO) represents an important predictor of heart failure progression and poor outcomes in HCM, especially in females [21]. Therefore, provocative maneuvers are mandatory in all patients, especially symptomatic patients without significant obstructions at rest [13]. In addition to gradient provocation, exercise echocardiography is recommended in all symptomatic patients with resting intraventricular gradients of <50 mmHg or in asymptomatic patients when provocation is relevant for their management and for risk stratification [13,22]. 

Accelerated color flow Doppler imaging helps to detect the presence of obstruction, and continuous-wave Doppler imaging is useful in measuring the peak gradient. In cases with turbulence of the color Doppler flow in the LVOT, it is recommended to interrogate the flow using a pulse-wave Doppler, starting in the LV apex or mid-ventricle and advancing the velocity sample box toward the LVOT and aortic valve to confirm aliasing of velocities at the LVOT level. The Doppler envelope typically has a late systolic peak or a mid-systolic temporary drop in cases of more severe obstruction. Care should be taken to avoid the mitral regurgitation (MR) jet velocity, which can overestimate obstruction severity [13]. The duration and shape of the jet should be considered to avoid measuring the MR jet instead of the obstruction flow. The MR jet starts with isovolumic relaxation, resulting in a longer systolic ejection period than the LVOT profile. The MR jet envelope typically has a mid-systolic peak and a more rounded appearance, with a peak velocity of about ≥6 m/s. A continuous sweep from LVOT flow toward MR flow can differentiate the two different Doppler profiles, as the flows will be displayed almost simultaneously adjacent.

The mechanism of obstruction is multifactorial. The obstruction was initially considered to be related to systolic contraction of the hypertrophied basal ventricular septum, which would then encroach into the LVOT with a resultant suction, or Venturi force, which would pull the mitral valve leaflets into the LVOT and produce further obstruction. Although isolated basal ventricular septal bulge is fairly common in older patients and can cause LVOTO, a ventricular septal bulge alone is usually not a significant cause of LVOTO. Rather, concomitant abnormalities of the mitral valve apparatus leading to SAM of the mitral valve play a central role by narrowing the LVOT [23]. Recent advances in imaging modality have enabled a deeper understanding of the complex way that the mitral valve apparatus contributes to dynamic LVOT obstruction. 

**Figure 4:**
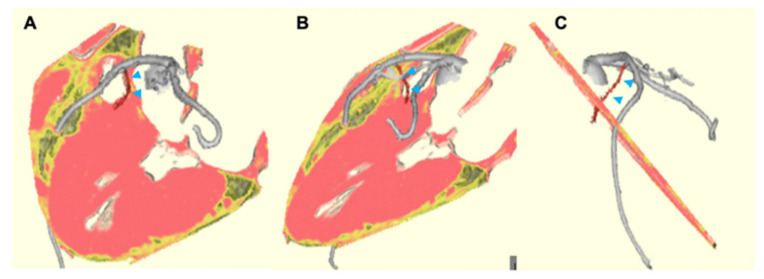
Three-dimensional CT images for the assessment of appropriate septal branch coronary artery for patients with significant left ventricular outflow tract obstruction planned for PTSMA. The arrowheads indicate the septal branch. CT = computed tomography, PTSMA = percutaneous transluminal septal myocardial alcohol ablation. D-dimensional views of the first major septal branch from different angles (**A**–**C**).

**Figure 5:**
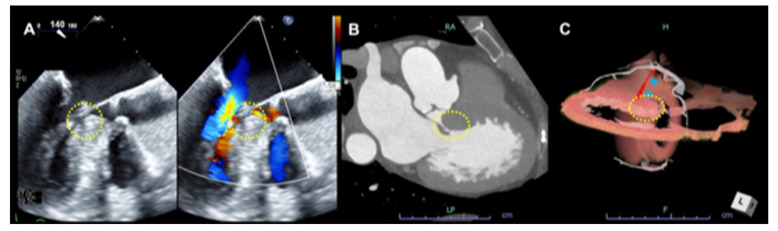
**A representative case of LVOT obstruction.** Transesophageal echocardiography (**A**), CT images for LVOT obstruction (**B**), and a target septal branch for PTSMA (**C**).

Significant LVOTO is another risk factor for symptoms, progression of heart failure, and mortality in patients with HCM [19]. Hemodynamically significant LVOTO is considered as a gradient of ≥50 mmHg. Although pharmacological therapy is effective to improve symptoms and reduce pressure gradient, residual LVOTO > 50 mmHg with drug-refractory symptoms is considered to be an indication for invasive septal reduction therapies, including surgical myectomy or percutaneous transluminal septal myocardial ablation (PTSMA) [1,8]. Although clinical outcomes are comparable between PTSMA and surgical septal myectomy, the choice of septal reduction therapy should be individualized based on the patient’s age, surgical risk, anatomical consideration, and extent of the hypertrophy, in addition to the patient’s preference [24]. Progress in cardiac imaging has provided useful intervention strategies in cases of PTSMA (Figure 4 and Figure 5). In particular, the importance of CT in the diagnosis and management of HCM has been increasingly recognized. In addition to the intervention strategies for PTSMA, advanced CT imaging can offer evaluation of coronary artery disease, distribution of LV hypertrophy, presence or absence of LVOTO or MVO, and left ventricular apical aneurysm.

### 3.2. Systolic Anterior Motion (SAM) of the Mitral Valve and Anomaly in the Mitral Valve Apparatus

One of the morphological features observed in the mitral valve apparatus that contributes to LVOTO is the SAM of the mitral valve [14]. The detection of SAM is classically done by M-mode echocardiography findings such as mid-systolic notching of the aortic valve and contact of the anterior mitral valve to the basal ventricular septum. There is less traction on the anterior leaflet, which is pulled into the LVOT in early systole by drag forces generated by the LV, thereby causing obstruction [25]. In the presence of SAM, failure of mitral valve leaflet coaptation can cause MR directing laterally and posteriorly. 

The severity of SAM is defined as mild when there is no mitral leaflet-septal contact, with a 10-mm minimum distance between the mitral valve and the ventricular septum. Severe SAM is defined as mitral leaflet-septal contact of >30%. SAM and LVOT obstruction are less common in other causes of LV hypertrophy, such as hypertensive heart disease and amyloidosis, which suggests that primary abnormalities of the mitral valve, specific to HCM, predispose individuals to develop SAM and LVOT obstruction. The displaced papillary muscle is typically hypertrophied and is compounding the obstruction by causing greater LVOT area reduction [1,26].

In patients with HCM, the mitral valve leaflet may be intrinsically normal. Rather, there may be abnormalities in the surrounding structures in the mitral valve apparatus, including elongation of the mitral chordae and anterior displacement of hypertrophied papillary muscles. Prominent displacement of papillary muscle directly into the mitral valve leaflet can be a cause of LVOTO. In the initial screening echocardiography for HCM patients, assessment of the mitral valve apparatus should be focused; leaflet morphology and coaptation, the severity of mitral regurgitation (MR), and the regurgitant jet direction should all be assessed. In patients with SAM, MR often coexists and is directed laterally and posteriorly. MR due to SAM predominates during mid- and late systole, and the severity of MR is proportional to the ventricular load and LV contractility that affect the severity of LVOTO. However, it is important to also diagnose other causes of mitral regurgitation, such as degenerative mitral valve prolapse, especially in cases with consideration of surgical or catheter intervention. 

### 3.3. Mid-Ventricular Obstruction (MVO) and Left Ventricular Apical Aneurysm (LVAA)

The site of obstruction is usually located in the LVOT, but it can also be present in the mid-ventricle or close to the apex due to the hypertrophied LV wall and/or papillary muscles abutting against the septum. Similar to the detection of LVOTO, color Doppler and pulse-wave Doppler are often used to identify the anatomic site of obstruction, and careful assessments of the whole LV (apex/mid-ventricular/LVOT) should be routinely conducted [13,14]. The gradient due to MVO also has significant variability, which relates to changes in loading conditions and contractility [27]. Significant obstruction can be detected only after provocation. The presence of MVO increases the risk of left ventricular apical aneurysms (LVAAs). 

**Figure 6:**
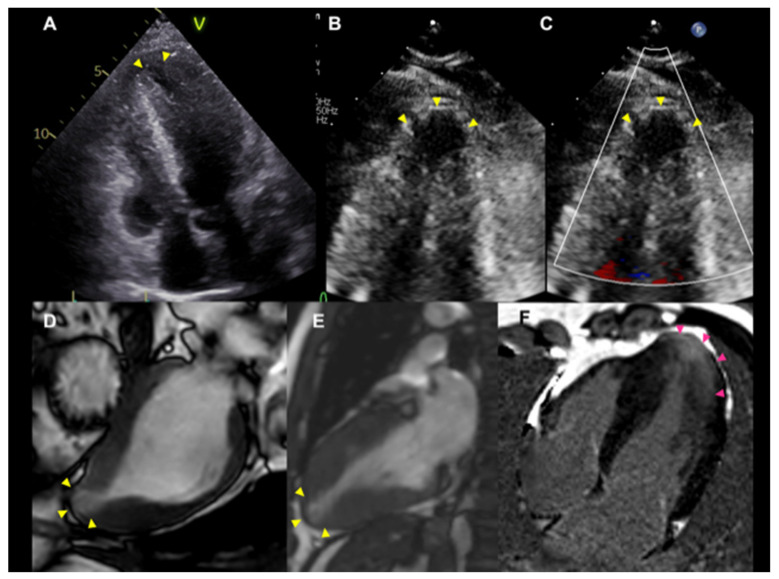
**Representative cases with left ventricular apical aneurysm.** Transthoracic echocardiography (**A**–**C**) and cardiac magnetic resonance imaging (**D**–**F**). Yellow arrowheads indicate apical aneurysms and red arrowheads indicate late gadolinium enhancement.

Patients with HCM and LVAAs are at risk for SCD or ventricular arrhythmias, and thromboembolic events in cases of apical thrombus. HCM with LVAAs larger than 4 cm is reported to be associated with increased risk of SCD, with a mortality rate of 3.4% per year [28]. Aneurysms can be visualized by echocardiography, but CT or MRI are particularly useful for the detection of apical aneurysms and thrombi (Figure 6) [13,14].

### 3.4. Reduced LV Function (Dilated Phase/End-Stage/Advanced Stage)

The clinical course of HCM is heterogeneous. Compared with the increased understanding of SCD risk stratification strategies, the risk factor of progressive heart failure remains unknown, although heart failure is responsible for approximately 60% of HCM-related deaths [29,30].

Left ventricular ejection fraction (LVEF) is typically preserved or supranormal in patients with HCM. However, LVEF is inadequate to evaluate systolic function in HCM. Patients with HCM usually have small LVs and reduced LV volumes, and LVEF can overestimate systolic function. A significant impairment of longitudinal LV systolic function is reported in patients with HCM who had preserved LVEF [31]. In a study including more than 3000 patients with HCM, abnormal global longitudinal strain (GLS) was associated with ventricular arrythmia [32]. Therefore, LVEF only decreases in the late-stage in a small subset of patients, less than approximately 15% [8]; this stage is referred to as the dilated phase, advanced stage, or end-stage [33,34,35]. An LVEF value of <50% indicates the end-stage or dilated phase of HCM [36,37], but data regarding optimal LVEF cut-off values for defining end-stage HCM are scarce. Extensive late gadolinium enhancement (LGE), defined as ≥15% of total LV mass, is reported to indicate the progression to end-stage HCM [38]. Nevertheless, longitudinal follow-up studies to identify risk factors and characteristics related to adverse LV remodeling are still warranted.

## 4. Risk of Sudden Cardiac Death

HCM is generally a low-event-rate disease, but SCD represents the most devastating complication of its natural history. It has been reported that the estimated overall incidence of SCD is 0.5–1.0% per year, compared with 0.2% in the general population, 2.0–3.0% in high-risk patients, including carriers of complex genotypes, and 4.7% in the presence of LV apical aneurysms [39]. Potential mediators for SCD include myocardial fibrosis, microvascular ischemia, and extensive disarray with disorganized myocyte arrangement. At present, there is no evidence-based pharmacological treatment for SCD prevention in HCM; the implantable cardioverter defibrillator (ICD) represents the only effective treatment for reducing SCD risk in high-risk individuals [1,8,40]. Therefore, risk stratification is important to assess those at risk of SCD; however, it remains challenging [41,42].

### 4.1. Risk Scores

The HCM Risk-SCD is a clinical risk prediction model for SCD based on a cohort of 3675 patients with HCM, providing 5-year risk analyses of SCD [43]. The HCM Risk-SCD uses seven variables: maximum LV wall thickness, LA size, LVOT gradient at rest as a continuum in addition to age, family history of SCD, non-sustained ventricular tachycardia (NSVT), and unexplained syncope. The 2014 ESC guidelines incorporated the HCM Risk-SCD model to classify patients as low-risk (5-year risk of SCD, <4%), intermediate-risk (5-year risk of SCD, 4–6%), or high-risk (5-year risk of SCD, >6%). ICD implantation was recommended as class IIB or IIA in the aforementioned groups. 

In a validation study of 1629 patients with HCM in the United States, the risk of SCD calculated by the ESC risk score was significantly lower than the actual event rate. Only 11% of 35 patients who had an SCD event were considered high-risk, which indicated that ICD was recommended, whereas 60% of the patients who experienced a clinical SCD event were low-risk, and ICD was not recommended [44,45]. On the other hand, Nakagawa et al. validated the Risk-SCD model in 370 Japanese patients with HCM and reported the supportive results, in particular for those with LVEF ≥ 50% [46]. In addition, a recent meta-analysis of 7291 patients showed that the prevalence of SCD endpoints was about 1% in low-risk patients, 2.4% in intermediate-risk patients, and 8.4% in high-risk patients, according to the HCM Risk-SCD score groups. Most SCD endpoints (68%) occurred in patients with an estimated 5-year risk of ≥4% [47], suggesting that the HCM Risk-SCD model provided reasonable risk estimations able to guide ICD therapy. However, there are no existing randomized trials or statistically validated prospective prediction models that guide ICD implantation in patients with HCM so far. In addition to the aforementioned risk scores, some HCM patients begin in an intermediate-risk group based on this current risk stratification, and additional echocardiographic parameters may be used to determine ICD implantation as a primary prevention. For example, patients with severe LV hypertrophy (MWT >30 mm) have a 3-fold higher risk for ventricular arrhythmia [9,48], and LVOT obstruction at rest increases the absolute risk of SCD from 0.9% to 1.5% [49]. Additionally, LV apical aneurysm, disarray, and myocardial fibrosis might be valuable to include in the risk score assessment for SCD.

### 4.2. Role of Cardiac MRI for SCD Risk Stratification 

While echocardiography is the first-line imaging modality in providing anatomical and functional details in patients with HCM, cardiac MRI (CMR) can often improve both the diagnosis and prognostic stratification, especially when echocardiography images are inadequate. CMR can provide excellent anatomical data regarding the distribution of myocardial fibrosis, assessed as LGE and extracellular volume [13]. Several studies have assessed the positive correlation between LGE and risk of SCD [38,50]. LGE exceeding 15% of the whole LV has been associated with a 2-fold increased risk in low-risk patients [51]. A combined use of global extracellular volume and ESC-SCD risk score has been reported to have better performance than LGE [52]. Additionally, CMR may help to detect right ventricular hypertrophy in HCM [53]. CMR can also provide a more detailed assessment of the multiple abnormalities in the mitral valve complex, including papillary muscle and chordal morphology and anatomy [54]. The information from the combined use of echocardiography and CMR is particularly useful to enhance our understanding of the mechanism of SAM and LVOT obstruction, leading to a better strategy for patients who are candidates for invasive septal reduction therapy. The risk of microvascular dysfunction has been suggested to be attributable to hypertrophy in HCM. Stress-induced perfusion detected by CMR or myocardial perfusion imaging-gated SPECT images are reported to be useful to detect microvascular disruption in patients with HCM [55,56].

### 4.3. Novel Echocardiographic Technique

Novel echocardiographic techniques are also useful to differentiate HCM from other causes of LV hypertrophy and to identify patients at high-risk of SCD or development of HF. Three-dimensional (3D) echocardiography has some advantages over standard 2D echocardiography, offering more information, such as the distribution and the extent of hypertrophy, the quantitative LV wall mass, and the mechanism of dynamic LV obstruction [14]. Furthermore, LV volumes, mass, and ejection fraction derived from 3D echocardiography have a stronger correlation than those obtained using CMR [13]. 

Myocardial deformation imaging can offer additional prognostic implications. A significant reduction in LV systolic velocity by tissue Doppler imaging (TDI) (<4 cm/s at the lateral site) has been reported as an independent predictor of death or hospitalization for worsening heart failure [57]. In addition, impairment of LV-GLS was associated with poor cardiovascular outcomes [32].

Speckle-tracking strain echocardiography can be used to assess mechanical and/or electrical dispersion, which may reflect heterogeneous contraction. Mechanical dispersion is defined as the standard deviation of time from Q/R wave onset on an electrocardiogram (ECG) to the peak negative strain in 16 LV segments [58]. A recent study has reported that mechanical dispersion was associated with malignant ventricular arrhythmias in cardiomyopathies and relates to fibrosis detected by CMR in patients with HCM [59,60,61,62]. 

## 5. Role of Pharmacological Therapy

Pharmacological interventions currently do not directly target the core molecular mechanism of HCM but are recommended to control symptoms, intraventricular pressure gradient, arrhythmia, and/or anticoagulation if needed [1,8].

Non-vasodilating β-blockers, such as bisoprolol, metoprolol, atenolol, propranolol, and nadolol, are usually the first-line therapy in symptomatic obstructive HCM [40]. Particularly, β-blockers are effective in patients with latent obstruction, although those with resting obstruction also respond [63]. β-blockers also have anti-arrhythmic effects. Specifically, propranolol was associated with a reduction in mortality in the pediatric population, especially in high doses [64]. In another study, sotalol was associated with a reduction in suppressing supraventricular and ventricular arrhythmias [65]. Calcium channel blocker (CCB) is sometimes used as an alternative in patients who are intolerant or have contraindications to β-blockers, with a class IB for verapamil and IIA for diltiazem. As the second-line agent, some class IA antiarrhythmic drugs, such as disopyramide or cibenzoline, can be an option when non-vasodilating β-blockers or verapamil therapy is ineffective. These drugs have negative inotropic effects and can decrease resting and provoked LV obstruction [40,66,67]. However, anticholinergic side effects may limit their use, and their efficacy may decrease over time. 

Amiodarone is employed as an arrhythmic control agent to reduce ventricular arrhythmic burden as well as the ICD shocks, often in combination with β-blockers. Drugs such as dihydropyridine CCB, nitrates, digoxin, angiotensin-converting enzyme (ACE)-inhibitors, and angiotensin II receptor blockers (ARBs) have risks associated with worsening intraventricular gradient, and it is recommended that they be avoided in patients with HOCM. 

### New Drugs 

The aforementioned traditional medical therapy is known to be effective in relieving symptoms; however, there is no evidence for any improvement in the natural course of the disease progression. Therefore, considerable efforts have been made to develop molecular approaches aimed at the core mechanism of the disease. Recently, a new pharmacological treatment was developed using a novel myosin inhibitor, mavacamten. This treatment was designed to reversibly inhibit β-myosin from binding to actin and to promote the super-relaxed conformation [68]; its effect includes an improvement in clinical outcomes, as well as a reduction of morbidity and mortality. In a phase-2 PIONEER-HCM study, mavacamten was associated with significantly decreased LVOT pressure gradient, increased peak VO_2_, and symptomatic benefits in patients with HCM [69]. A recent phase-3 EXPLORER-HCM multicenter, double-blind, placebo-controlled study evaluated the efficacy and safety of mavacamten in 251 symptomatic patients with obstructive HCM. Patients were randomly assigned to oral mavacamten or a matching placebo once daily for 30 weeks. Dose titration was individualized from 2.5 mg to 15 mg to achieve the target reduction in the LVOT gradient (<30 mmHg) and the target mavacamten plasma concentration (350–700 ng/mL). Patients who received mavacamten were associated with an increase in peak VO_2_ (≥3.0 mL/kg/min) or peak VO_2_ (≥1.5 mL/kg/min), with NYHA improvement as a primary endpoint (37% vs. 17%, *p* = 0.005). In addition, patients treated with mavacamten were associated with reduced LVOT gradient and improved heart failure biomarkers, symptoms, exercise performance, and health status [70]. Mavacamten was associated with reduced LV wall thickness and LA volumes, and it improved diastolic parameters compared with baseline [71]. Based on these promising results, mavacamten has a potential to change the clinical practice in the management of HCM. 

## 6. Role of Genetic Testing

HCM is one the most common genetic cardiovascular diseases. At present, more than 1400 mutations in 11 or more genes have been identified in patients with HCM, with the majority encoding proteins of the cardiac sarcomere. Affected genes include β-myosin heavy chain (*MYH*), myosin-binding protein C (*MYBPC3*), cardiac troponin T (*TNNT2*) and I (*TNNI3*), α-tropomyosin (*TPM1*), actin (*ACTC1*), titin (*TTN*), and myosin light chains (*MYL3*) [14]. Types of mutations vary and include deletions, insertions, missense, and splice site mutations. These known mutations are inherited in an autosomal dominant manner. However, not all HCM patients have detectable pathogenic mutations, and mutations in other genes outside of the sarcomeric myofilaments have also been implicated in HCM. Previous studies have reported that patients with sarcomere mutations were associated with an increased risk of developing heart failure compared with patients with non-sarcomere mutation or negative results from genetic testing [72,73,74]. 

The identification of pathogenic mutations enables us to screen first-degree relatives who are at risk of developing HCM. Although several studies have reported that genotype-positive relatives without LV hypertrophy had subtle changes in myocardial function or the mitral valve apparatus [75], the clinical course of these patients is favorable. In a recent series of 203 individuals, only 10% converted to a true HCM phenotype, and none experienced cardiac events [76]. In another study from the Framingham and Jackson Heart Study cohorts, 11.2% of participants with sarcomere gene variants carried at least one rare non-synonymous sarcomere variant, and 0.6% of participants carried mutations considered to be pathogenic based on conservative criteria [77]. Interestingly, many of these participants were not diagnosed with HCM, although the sarcomere variants indicated an increased risk of future cardiovascular events [77]. 

## 7. Conclusions and Future Perspective

Over the past 20 years, the pathophysiological understanding and clinical management of HCM have changed significantly. In particular, the introduction of ICD for SCD prevention is a paradigm for disease management. Although the SCD-risk model is proposed by existing guidelines, more precise risk stratification models are still needed. In addition to prevention for SCD, risk stratification for progression of heart failure or dilated HCM remains inconclusive. 

The combined use of novel imaging techniques has the potential to identify several structural or functional abnormalities related to adverse events in HCM, leading to future gene-directed approaches, as well as assessment of the effect of novel pharmacological management on the disease progression and outcomes. In addition, further mechanistic studies are warranted for a deeper understanding of the phenotype–genotype linkage; such studies have the potential to lead to the development of novel specific therapeutic approaches for HCM.
